# Monitoring the Impact of Spaceflight on the Human Brain

**DOI:** 10.3390/life12071060

**Published:** 2022-07-15

**Authors:** Michael F. Dinatolo, Luchino Y. Cohen

**Affiliations:** 1 Department of Epidemiology and Biostatistics, Schulich School of Medicine and Dentistry, Western University, London, ON N6A 5C1, Canada; mdinatol@uwo.ca; 2 Canadian Space Agency, 6767 Airport Road, Saint-Hubert, QC J3Y 8Y9, Canada

**Keywords:** deep space, neuroimaging, brain monitoring, radiation, microgravity, isolation, brain, behavioral performance

## Abstract

Extended exposure to radiation, microgravity, and isolation during space exploration has significant physiological, structural, and psychosocial effects on astronauts, and particularly their central nervous system. To date, the use of brain monitoring techniques adopted on Earth in pre/post-spaceflight experimental protocols has proven to be valuable for investigating the effects of space travel on the brain. However, future (longer) deep space travel would require some brain function monitoring equipment to be also available for evaluating and monitoring brain health during spaceflight. Here, we describe the impact of spaceflight on the brain, the basic principles behind six brain function analysis technologies, their current use associated with spaceflight, and their potential for utilization during deep space exploration. We suggest that, while the use of magnetic resonance imaging (MRI), positron emission tomography (PET), and computerized tomography (CT) is limited to analog and pre/post-spaceflight studies on Earth, electroencephalography (EEG), functional near-infrared spectroscopy (fNIRS), and ultrasound are good candidates to be adapted for utilization in the context of deep space exploration.

## 1. Introduction

As the world looks toward the new frontier of deep space exploration, an important part of exploring this new environment will be protecting the health of astronauts (and space tourists) [1]. In this review, deep space is defined as the environment that exists outside of Earth’s magnetosphere. The brain has the potential to be afflicted by most of the main risks associated with deep space. Prolonged periods of microgravity exposure, increased levels of radiation, and long periods of isolation in extreme environments are all characteristic of this environment [2]. Neurological analysis technologies are vital in studying the effect of spaceflight on the brain and in monitoring neurological treatments of astronauts during space travel. They are essential to gain a better understanding of the impact of the deep space environment on the central nervous system (CNS) and to characterize and prevent neurological risks. These can vary from structural, functional, and behavioral effects resulting from exposure to this environment [2,3]. Here, we review the current knowledge on the neurological risks associated with the deep space environment, and the potential of brain monitoring technologies to be adopted in space, on Earth following a spaceflight, or in space analogs.

## 2. The Effects of Spaceflight on the Brain

Investigations on astronauts following missions to the International Space Station (ISS) have revealed that the human brain undergoes a variety of changes during spaceflight, and in deep space these changes will likely be accentuated [2,4]. They include micro- and macro-structural changes of grey matter, changes in cerebrospinal fluid (CSF) and fluid distribution, as well as changes in behavioral and cognitive performance [2,4,5]. For example, responses and actions controlled by the vestibular system have seen structural and functional changes at multiple stages of vestibular processing due to exposure to microgravity and simulated microgravity [6]. Deep space radiation will expose astronauts to higher levels of ionizing radiation, as compared to Low Earth Orbit (LEO), due to the higher linear energy transfer (LET) of the particles in deep space and the absence of Earth’s protective magnetic field [7]. This will be particularly relevant to the next phase of human space flight, the Lunar Gateway program, with a Moon-orbiting habitat that will support Moon surface missions. This comes with increased risk of both short-term and long-term negative health outcomes. Acute consequences of radiation exposure can range from negatively affected cognitive function to CNS damage or even death if astronauts are exposed to a very large quantity of particles [8]. Long-term effects of radiation are more concerning and can include increased risk of cancer development, or permanent damage to structures of the brain [2,8]. Developing methods of preventing radiation exposure or reducing it to safe limits is especially difficult for deep space. Future astronauts will need to be protected from this unique risk [9].

Although there are clinical data on radiation exposure and its effect on the human brain, total doses, dose rate, and radiation quality are very different in deep space [10]. Studies using animal models have demonstrated that exposure to simulated cosmic radiation triggers cognitive impairments in spatial memory and complex learning [10]. Some of this evidence also indicates the potential for mental health conditions, such as the manifestation of chronic anxiety [2,8]. Rodent models have also been used to investigate the effect of the radiation environment on structural and functional neural mechanisms. These studies have indicated the potential for wide-ranging radiation-induced structural, cognitive, and behavioral changes because of exposure to radiation at space-relevant fluency levels. Some functional behavioral deterioration can be attributed to the erosion of neuronal structure and synaptic integrity [2].

In addition to increased levels of radiation, exposure to microgravity will be another key consideration. Microgravity is known to influence physiological, structural, and functional processes in the human body [11]. For instance, long-duration spaceflight increases the total intracranial volume in the brain [12]. This also results in structural changes in the brain, as well as changes in the intracranial and intraocular pressure. Conditions such as Spaceflight-Associated Neuro-Ocular Syndrome (SANS), which was previously known as vision impairment and intracranial pressure (VIIP), likely result from increased intracranial pressure, which also causes cognitive impairments or effects on performance such as neurovestibular issues [2].

Prolonged periods of isolation beyond those that astronauts have experienced in LEO will be another key risk factor [13]. Due to the distance between the Lunar Gateway and Earth, astronauts will live in an isolated, confined, and extreme (ICE) environment, to a much larger extent than during ISS missions. Previous human studies in ICE environments, including analogs of the deep space environment, suggest that short-term and long-term effects will also differ from the ISS. These include impaired cognition, environmental triggers of the immune response, as well as psychological and physiological effects of the strenuous environment [14]. Long periods of isolation can result in several psychosocial stressors that have negative impacts on both performance and behavior [14]. Some studies show that exposure to ICE environments has negative impacts on sleep, which in turn causes additional stress and mental health challenges and affects performance to perform tasks during missions [15]. Risks associated with isolation are not limited to psychological or physiological effects, as structural changes were also shown to occur in the brain. Isolation studies such as the “Mars500” study were particularly useful to identify the impact of isolation on the CNS. A small group of volunteers were confined in an environment simulating a mission to Mars. They were isolated for a stunning 520 days, and using diffusion tensor imaging, researchers identified microstructural changes in the white matter of the brain [13]. Microstructural changes and damage in white matter have been associated with neurocognitive deficits in high-altitude pilot populations [5,16]. Long-term isolation in deep space can lead to a whole suite of long-lasting changes from a reduction in performance in the brain areas such as the sensorimotor network where microstructural changes are observed post-flight [17].

These studies have provided important insight into the effects of spaceflight on humans, but data obtained beyond the Low Earth Orbit are not yet available. In addition, findings in available studies are mainly based on pre- and post-flight measurements of structural, functional, and behavioral effects of spaceflight. Pre- and post-flight studies often have the disadvantage of simultaneous exposures to several risk factors [18]. In-flight monitoring technologies would enable investigators to measure changes in the CNS while in the deep space environment and reduce the possibility for bias or influence by other factors (i.e., changes associated with orthostatic intolerance) upon leaving the deep space environment [19,20]. In addition, a delay in the imaging of the brain post-exposure can have a significant impact on the estimation of the true effect of deep space on the brain [17]. Several candidates for technologies for brain function monitoring applicable to spaceflight are described below.

## 3. Monitoring Technologies Adaptable to Spaceflight

A total of three technologies that can be used during spaceflight were identified in the scientific literature. These technologies have or can support research conducted in the Low Earth Orbit (LEO).

### 3.1. Electroencephalogram (EEG)

EEG is a non-invasive technology that records electrical activity across the cerebral cortex. By measuring activity at the synapses of firing neurons, called postsynaptic potentials, the electrodes receive the information needed to produce an electrical map of the brain [21,22]. This map represents the different frequencies of brain waves that each have unique characteristics and diagnostic uses for participants. Spectral content is divided into four frequency bands: delta (0.5–4 Hz), theta (4.25–8 Hz), alpha (8.25–13 Hz), and beta (13.25–32 Hz). Signals from different frequency bands reflect different neurological phenomena. Each signal band will also have a typical wave pattern and abnormal signals will indicate issues in brain activity [23]. It is through comparing typical signal patterns with individuals exposed to various risk factors that researchers can determine etiological factors for changes in performance or detect neurological disorders.

**Figure 1:**
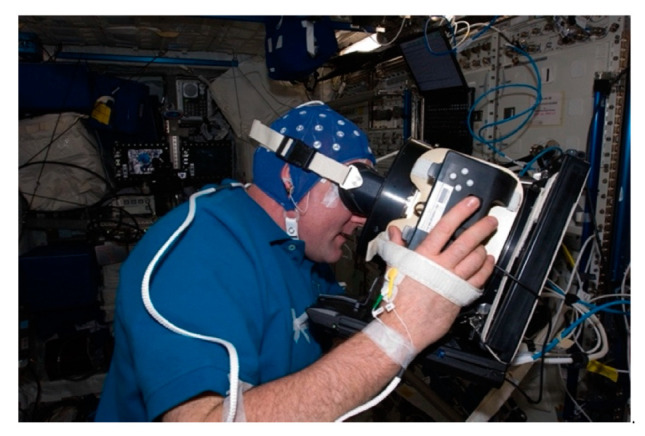
**Data collection with an EEG electrode cap onboard the ISS**. European Space Agency (ESA) astronaut Andre Kuipers is wearing an EEG electrode cap for the NEUROSPAT investigation. (NASA Image: ISS030E022613).

EEG will be essential for studying the unique risks of deep space on the CNS. On Earth, EEG can highlight abnormal brain activity due to epilepsy, head injury, stroke, or even brain tumors [24,25,26,27]. The “NEUROSPAT” project has demonstrated the potential of this technology on the ISS (Figure 1). It has confirmed changes in visuo-attentional activity, visuospatial performance, brain activity, and effects of space travel on sleep quantity [28,29,30,31]. EEG has also been used in space to measure changes in neurocognitive performance and brain activity that result from exposure to microgravity and isolation [27]. Changes in activity are commonly identified in the faster EEG frequencies (alpha and beta). Simulated microgravity environments, specifically during bed rest, see changes in theta and alpha waves, demonstrating inhibition of brain activity in the cortical regions under these conditions. Many of the changes in EEG activity may result from reduced sensorimotor input in simulated microgravity [32]. Findings also point to reduced sleep duration and slow-wave sleep (SWS) during spaceflight due to isolation and microgravity. Performance reductions are another issue in microgravity environments. Docking simulation tasks indicate an increase in global theta EEG oscillations while in space as compared to the same task on Earth. This was also associated with slower reaction times during the task, which has important implications for astronauts [33].

Spaceflight analogs, such as parabolic flights and terrestrial studies, have also utilized this device. Studies of reaction time have demonstrated that executive functioning arithmetic tasks can be affected by exposure to small periods of microgravity. In general, reaction time increased as a function of task complexity, although with the most complex levels, reaction time was significantly reduced in microgravity conditions [34]. The superior frontal and medial frontal gyrus in microgravity may be involved in the reduction of certain waveform amplitudes when measuring EEGs of participants. Surprisingly, cortical processes are enhanced during short exposure to microgravity in parabolic flights, which suggests that previously reported impairments in cognitive performance are likely attributable to increased stress rather than weightlessness itself [34]. In analog studies on Earth, adopting a less upright posture leads to increases in thoracic blood volume and baroreceptor stimulation (similar to what is seen in microgravity) and to cortical activity being inhibited when reclining or tilted head-down. Similarly, in zero gravity, there is a redistribution of blood towards the upper body that stimulates baroreceptors. This stimulation is associated with an inhibition of cortical activity during zero gravity, and parabolic flight [35]. Due to the multifaceted and complex relationship that inhabited activity across cortical regions may indicate, more research must be carried out to determine the effects of increased cortical inhibition on performance, and the development of psychological disorders. Analog studies on Earth using head-down bed rest as a proxy for microgravity’s effect on fluid distribution have led to increased incidence of anxiety and depression in participants, however this has not yet been connected to cortical inhibition [36].

To assess the impacts of radiation, similar studies using EEG have been performed with radiotherapy patients who exhibit chronic fatigue, depression, and other behavioral changes. However, these patients were not exposed to the same type of radiation present in deep space. EEG will be useful for medical operations via its diagnostic capabilities, although more research will be required to establish its future as a medical device for deep space [25]. One potential application related to the deep space environment could be detecting neuronal brain tumors. These types of tumors are identified by observing continuous spiking in EEG wave patterns. Neuronal tumors are commonly present with seizures and EEG can be an important tool for determining the cause of a seizure during a mission [25].

Many microgravity- and isolation-related afflictions can be diagnosed and therefore treated early using brain monitoring. Brain health monitoring using EEG can establish longitudinal baselines for astronauts in various health metrics and even vital signs [37,38]. Cognitive functions and mood states are also affected in microgravity environments and their analogs, and these effects can be monitored using EEG [36]. Long-duration spaceflight, as mentioned earlier, introduces new stressors due to the longer exposure to isolation, microgravity, and radiation. EEG assessments for these disorders are performed clinically and are relevant to the deep space environment.

### 3.2. Functional Near-Infrared Spectroscopy (fNIRS)

The fNIRS technology is a non-invasive optical imaging technique that measures changes in hemoglobin (Hb) concentrations in the brain. It takes advantage of the absorption spectra of Hb in the near-infrared range [39]. The difference in light absorption is used to assess the concentration of Hb in a particular region, which acts as a measure of cerebral activity. This technology can be used for functional neuroimaging, as well as cerebral oximetry, diffuse optical tomography, and hyper-scanning. The fNIRS is used to quantify oxygen concentrations, Hb concentrations, blood oxygen level-dependent (BOLD) signal, and functional connectivity [39].

Although this technology has not yet been used in space, it has been used in parabolic flights for real-time monitoring of attentional states. Studies using real-time task monitoring with fNIRS have shown that the default mode network, which is a resting state functional network, has lower activity during goal-oriented behavior [40]. These activities occur at rest, or in the absence of tasks [41]. This allows for the identification of attentional lapses and can uncover associations with poor performance due to sleep deprivation. There have also been findings of frontal brain oxygenation being sensitive to workload during complex tasks, but not necessarily predictive of a decrease in performance [40].

The fNIRS system is a highly portable, a relatively inexpensive, and a lower power version of an fMRI system. For tasks in which safety is critical, an fNIRS system can use information on cerebral activity to provide insight into the decision making of the astronaut, and the quality of the decisions being made. In addition, fNIRS can be used in functional brain imaging for studies that would typically use an MRI, which makes it an invaluable tool in studying deep space risks commonly investigated by MRI [42]. This technology could support studies on decision-making, attention, and behavior in performance- or task-based environments in the context of spaceflight. Areas of research such as fatigue, sleep deprivation, neurocognitive performance, and social cognition (especially through the lens of multiple brain hyper-scanning) are all evolving areas of fNIRS research [43]. These research areas directly lead to a wide range of operational- and research-related applications for the deep space environment’s strenuous ICE conditions. Interestingly, an integrated fNIRS/EEG system has been developed. This hybrid system can acquire and analyze multiple brain signals and provide a comprehensive picture of brain function that cannot be obtained with only one of the components. Combining two methods that represent two different physiological processes (neuronal electrical activity and the hemodynamic activity of the brain) allows to draw conclusions from the simultaneous real-time data [44].

### 3.3. Ultrasound

Ultrasound is an imaging technique that uses high-frequency soundwaves to produce images of structures in the body. Upon interacting with these structures, the soundwaves bounce off the internal body structures and create an echo used to produce the images. These echo waves are turned into electric signals and are converted into an image [45]. This type of device has been used in analog environments to quantify middle cerebral artery velocity (MCAv), commonly reported in cm/s, which is important in measuring intracranial pressure and intracranial volumetric ratios [46].

**Figure 2:**
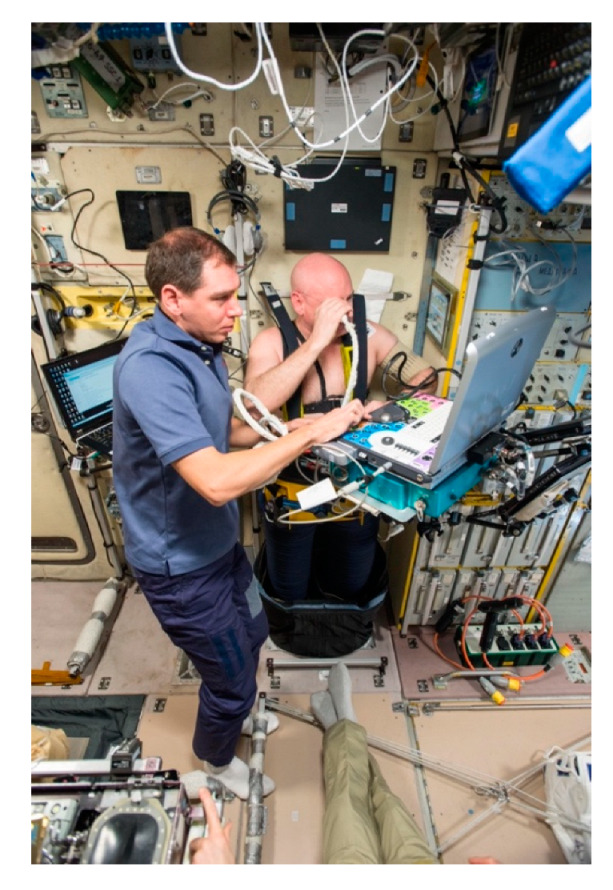
**Ultrasound onboard the ISS for measuring fluid shifts**. Ultrasound for fluid shift experiments performed on NASA astronaut Scott Kelly (NASA Image: ISS045E015549).

Ultrasound could be a useful tool for understanding the risks astronauts face during microgravity exposure due to fluid shift and can also be applicable to longer bouts of exposure in deep space (Figure 2) [46,47,48]. Exposure to microgravity results in blood volume redistribution and blood pooling in the upper body, which leads to increased intracranial pressure [49]. Due to its ability to perform velocity- and pressure-based measurements, ultrasound can be used to quantify key metrics such as pressure and velocity of blood flowing to the brain. Onboard the ISS, ultrasound has been used to assess relationships between fluid shifts, intracranial pressure, visual impairments, and cerebral blood flow. The “Drain Brain” study, for instance, monitored the cerebral outflow of astronauts onboard the ISS and used ultrasound to measure the cross-sectional area of the internal jugular vein (IJV), allowing for the study of changes in the fluid distribution, from the lower body to the upper body specifically [50]. Other studies have measured the dilation of the optic nerve with ultrasound, using it as an index of intracranial pressure in both spaceflights and analog studies. SANS research using analog environments has also investigated microgravity fluid shifts as the cause of mild thickening of the retinal nerve fiber layer [51]. The mechanisms of SANS involving fluid shift remain ill-defined, but ultrasound has the potential to further investigate the relationship between optic nerve swelling and increase pressure from fluid shift towards the brain [47,52].

## 4. Monitoring Technologies Not Suitable for the Spaceflight Environment

### 4.1. Magnetic Resonance Imaging

Magnetic resonance imaging (MRI) is a widely used neuroimaging technology that takes advantage of the different concentrations of hydrogen atoms found within the human body, specifically in water and fat, to produce images of the human brain, or any other part of the body [53]. MRI can be used for structural imaging, brain activity monitoring, diffusion imaging, and overall diagnostic purposes [53,54]. It is a non-invasive imaging technology that produces three-dimensional detailed anatomical images through the imaging of grey and white matter tissue of the brain. These images are made up of three-dimensional pixels, called voxels. Larger voxels will result in a lower-resolution image, while smaller ones will afford higher spatial resolution images. Spatial resolution is dependent on the size of the matrix, field of view, and the slice thickness [55]. Larger voxels improve the signal-to-noise ratio, but support a lower spatial resolution, while smaller voxels increase spatial resolution and worsen the signal-to-noise ratio or temporal resolution. T1 images tend to require higher spatial resolution, and T2 a higher temporal resolution [55,56]. MRI signals can provide a wide variety of information on the CNS, using one of the following modes.

#### 4.1.1. Structural Magnetic Resonance Imaging (MRI)

**Figure 3:**
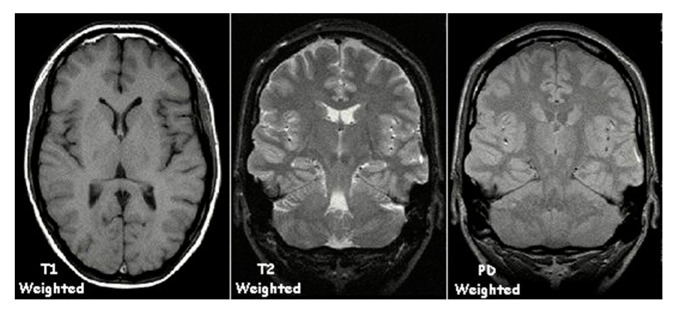
**T1-weighted, T2-weighted, and proton density-weighted MRI images**. Reproduced from https://en.wikipedia.org/wiki/Magnetic_resonance_imaging#/media/File:T1t2PD.jpg, accessed on 24 April 2022.

MRI images are typically performed using one of three separate imaging contrasts: proton density imaging, T1 imaging, and T2 imaging (Figure 3). Proton density imaging is a measure of the signal derived from the protons (mostly from the hydrogen of water molecules) in the brain. Areas with a high concentration of hydrogen atoms will appear as white in these images, while low concentrations of hydrogen will be darker or grey in comparison [57]. T1-weighted images are obtained in the same manner as the original proton density image, however the scan uses a sequence of radio frequency (rf) pulses that can identify fat in the body, such as the myelin sheet that surrounds the axon, as a bright white, while water molecules and cerebral spinal fluid (CSF) are darker on the image. These sorts of images are best for structural studies as a clear contrast exists between the white matter and the grey matter. Finally, T2-weighted images identify water as bright white in the image and fat as darker. This can highlight the movement of water or CSF throughout the brain more easily and is often utilized in functional imaging [58,59]. MRI also measures brain metrics such as volumes, surface areas, regional thickness, and other measurements that characterize the structural features of the brain [60].

Standard MRI has been useful in pre- and post-flight studies or in analog environments to assess structural changes in the brain. Reduction in cortical region thickness, intracranial pressure changes, and volumetric changes of white and grey matter are all examples of the anatomical effects of the spaceflight environment assessed with structural MRI [60]. Long-term microgravity exposure causes expansion of the brain and increases in cerebrospinal fluid (CSF) volumes due to white matter and lateral ventricular changes. Measurements stayed elevated one year after spaceflight, suggesting long-term effects. An increase in mean CSF intraventricular (aqueduct) flow velocity, and depression of the pituitary dome compared with baseline, are also seen post-flight. Changes in the lateral ventricular volume and aqueduct CSF flow parameters were similar to those seen in individuals with normal pressure, communicating hydrocephalus [61]. An increase in the white matter volume of astronauts who return from a long-term spaceflight was also reported, with significantly higher levels of global white matter in the brain [4]. There are also observable upward anatomical shifts in the position of the brain and brain stem, as well as narrowing of CSF spaces in the brain. While in flight, this can manifest itself in hydrocephalus-like symptoms (i.e., reductions in cognitive performance) that do not persist long-term post-flight. Structural MRI has a wide range of applications in assessing the effects of the deep space environment on the brain.

#### 4.1.2. Functional Magnetic Resonance Imaging (fMRI)

**Figure 4:**
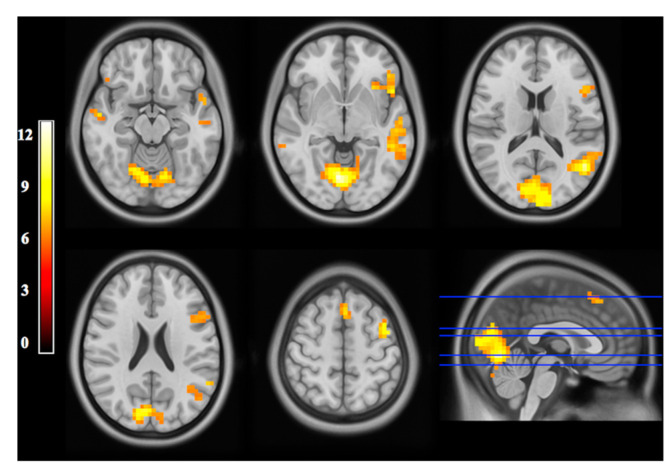
**Functional MRI image.** fMRI statistical maps of BOLD signal represented as axial slices. The bottom right image is a cluster-thresholder t-stat map. Doi:10.1371/journal.pone.0152614 (accessed on 24 April 2022).

Functional magnetic resonance imaging (fMRI) uses the increase in deoxygenated hemoglobin in the various regions of the brain as a proxy for brain function. This imaging utilizes T2*-weighted imaging, which detects heterogeneities or irregularities in the MR signal caused by the concentration of deoxyhemoglobin (HBR) [62]. When a region of the brain is activated, the hemodynamic response allows for the diversion of oxygen-rich blood to regions with increased activity. Therefore, regions with more oxygenated hemoglobin will have a higher level of signal received in addition to the signal received from hydrogen atoms. This process of linking local neuronal activity and the increase in local blood flow (oxyhemoglobin) is called neurovascular coupling, used as a proxy for neuronal activation. The scanner sends the electrical signal to a computer which converts it into a three-dimensional representation of brain activity detected during the scan (Figure 4) [63]. In the literature, the signal that the scanner measures is referred to as the blood-oxygen level-dependent, or BOLD, signal. In addition, a measure called functional connectivity is a useful way of correlating the BOLD signal across different brain regions or networks that are involved while performing a given task [64].

Although there are no examples of fMRI, or any MRI, being used during spaceflight, analog and pre- and post-flight studies of the space environment have produced informative findings using fMRI. They have confirmed significant changes in the functional connectivity of motor, somatosensory, and vestibular areas of the brain after 70 days of head-down bed rest (HDBR) [6,65]. Skull-tap vestibular stimulation results in activation of the vestibular cortex and deactivation of the cerebellar, motor, and somatosensory cortices. Increased activity of the bilateral insular cortex of the vestibular network increased during head-down bed rest. A strong response in the frontal, parietal, and occipital regions was associated with greater loss of balance and mobility after HDBR. This effect suggested reduced neural efficiency during functional mobility tasks as a result of HDBR. This only results in short-term reductions in functional connectivity which subside after HDBR, though neural efficiency has been correlated with differences in cognitive task performance between higher and lower intelligence individuals [6].

Many network alterations are significantly associated with changes in sensorimotor and spatial working memory performance [4,65]. Pre- and post-flight studies investigated the effects of the space environment on brain connectivity, performance, and behavioral changes during spaceflight. Network-based statistics demonstrated task-specific functional connectivity changes in regions with activation sites across parts of the sensorimotor neural network and the visual, proprioceptive, and vestibular systems [4,66]. Post-flight increases in stimulation-specific connectivity in the right posterior supramarginal gyrus, an increase in connectivity between left and right insulae, and a decreased connectivity of the vestibular nuclei, right inferior parietal cortex, and cerebellum were all identified [66]. Increased connectivity has also been identified in the right and left posterior insula and decreased connectivity between the posterior cerebellum and primary visual cortex. Other studies have shown reductions in intrinsic connectivity in the right insula and ventral posterior cingulate cortex and reduced connectivity between the right motor cortex and left cerebellum. These are findings that have been identified during resting state fMRI [67].

#### 4.1.3. Diffusion MRI (dMRI)

Diffusion MRI (dMRI) utilizes the MR signal received from water molecules to structurally visualize white and grey matter structures, and the diffusion of water in the brain [68]. The dMRI technique provides information on the structure of various white matter and grey matter regions in the brain, differentiating between isotropic and anisotropic diffusion. Isotropic diffusion is a type of unrestricted diffusion associated with grey matter and describes the random, spherical net movement of water in a region. Such water movement mimics a bubble expanding. Anisotropic (restricted) diffusion is seen in white matter regions and describes restricted movement of water in a non-random and non-spherical direction, following nervous fibers [54,68]. Diffusion-weighted imaging (DWI) codes anisotropic diffusion of water through the brain as a white voxel, and isotropic diffusion of water as a grey voxel. This produces an image of the brain that distinguishes grey matter and white matter, as well as areas of significant anisotropy, such as in patients with ischemic stroke [54,68]. DTI is a type of DWI that allows researchers to determine the three-dimensional direction of diffusion as water passes through neurons. DTI metrics define the brain structure and microstructure of the various white matter tracts in the brain. It can even produce images of the white matter tracts, visualized as bundles of neuron axons, within the brain [69].

DWI measures structural connectivity, or the structure and direction of diffusion, through the various grey matter and white matter tracts. DTI can additionally identify white matter microstructure using the degree of anisotropy in a region of the brain measured as the fractional anisotropy (FA) values. In addition, dMRI also measures mean diffusivity (MD), axial diffusivity (AD), and radial diffusivity (RD). Mean diffusivity is the average rate of diffusion in a specific region, axial diffusivity is the average rate of diffusion parallel to the axon, and radial diffusivity is the average rate of diffusion in the directions perpendicular to the axon. This is important when trying to identify diffusion inhibition, as it could imply abnormalities in the structure of the brain.

Spaceflight studies have revealed significant microstructural changes in several large white matter tracts, including the corpus callosum, arcuate fasciculus, corticospinal, corticostriatal, and cerebellar tracts [17]. In microgravity analog environments, the decrease in extracellular free water in the post-central gyrus and precuneus was negatively correlated with balance changes. The grey matter increase in these regions was associated with a reduction in bed rest-induced balance impairment [70]. There are even observable changes in the astronaut brain during and after a long period of isolation, as shown in the “Mars500” study. Significantly lower fractional anisotropy (FA) was found in participants as compared to controls. This was observed in the anterior parts of the callosal body. Furthermore, after confinement, widespread FA reduction could be seen in the right hemisphere and ending in the temporo-parietal junction zone. All areas with decreased FA predominantly showed an elevated radial diffusivity and mean diffusivity, while axial diffusivity was less correlated. Reduced fractional anisotropy in the right temporo-parietal junction could specifically indicate reduced prediction errors in visuospatial processing due to limited space and a partly stimulus-deprived environment over a long period. In addition, microstructure features (lower FA) in the anterior callosal body are negatively associated with intelligence [13].

### 4.2. Positron Emission Tomography (PET)

**Figure 5:**
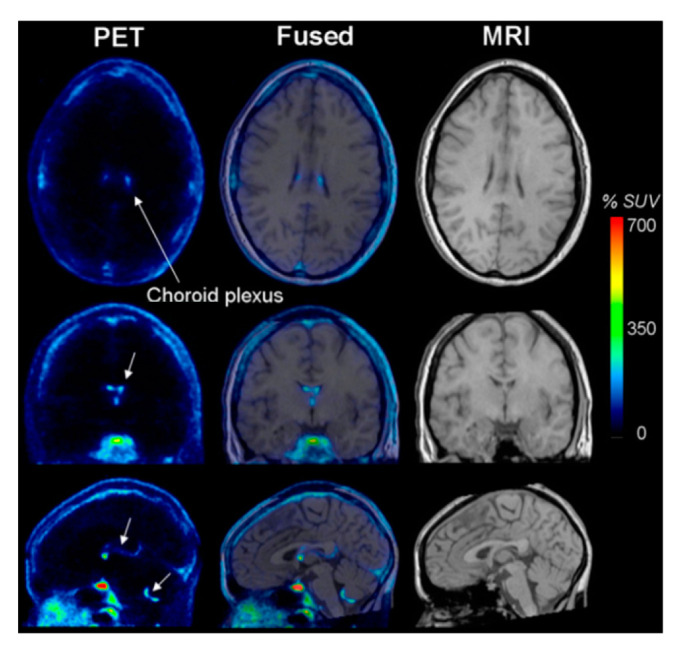
PET Image. Brain image taken using positron emission tomography (PET) and T1 MRI images. Red regions of the image show areas of tracer accumulation. The fused image is the PET image superimposed on the structural MRI image. http:/doi:10.2967/jnumed.108.058453, accessed on 24 April 2022.

PET measures cellular metabolic activity within the human body. PET takes advantage of radioactive tracer molecules that are glucose or oxygen analogs, which emit a positron to stabilize the compound [71]. These radiotracers circulate throughout the body in the blood stream, and can reflect metabolic activities, blood flow, and oxygen utilization. Activity is quantified and qualitatively visualized, with identified activity in an image corresponding to accumulations of radiotracer in brain areas with increased glucose or oxygen metabolism [72,73]. When the tracer converts to a stable molecule and emits a positron, the particle annihilates an electron and emits energy in the form of photons. Those photons are also known as gamma rays and are picked up by the scanner to produce an image that can identify the locations in which the radio tracer is found in the body (Figure 5) [71,72,73]. Single-photon emission-computed tomography (SPECT) is a simpler alternative that uses a similar mechanism, but only uses one positron, and therefore one gamma ray, to perform the imaging. Imaging with this technology is of a lower quality, but it offers a more affordable scan to consumers [73].

PET can offer some important information regarding astronaut health through post-flight data collection or after exposure to a risk factor observed in deep space [74]. It can be used to measure physiological activation and metabolism in the brain, unlike any other technology mentioned. PET can be used in situations that require monitoring of metabolism, blood flow, and physiological activity, such as neurotransmitter systems [71]. It is especially valuable in identifying areas in which glucose metabolism is abnormal or highly increased [75]. This technology can be used to identify cognitive performance changes via monitoring of the physiological environment and to identify suspected tumors in astronauts who have been exposed to larger amounts of radiation or smaller doses over long periods of time [71,75].

### 4.3. Computerized Tomography (CT)

CT is an imaging technique in which a series of two-dimensional X-ray scans are performed for the purpose of producing a 3D image of the desired part of the body. A CT scanner will deliver X-rays though the body, with the source and receiver rotating around the patient at 360 degrees. The receiver is opposite to the X-ray source to detect light that exits the body and constructs a 3D X-ray image with the signal [76]. As X-rays are a form of ionizing radiation, this can cause increased risks compared to other technologies [77].

Although CT systems cannot be launched aboard a space station due to their size, there have been mentions in the literature of using the technique as a pre-flight screening tool. A case report that discusses the case of a female astronaut who had experienced an arteriovenous rupture anomaly post-training for spaceflight highlights the utility of CT. Although it was unknown if her training led to the rupture, investigators raised the question of using CT as a screening mechanism for all astronauts before being cleared for a mission [78]. CT can produce 3D models of various structures in the brain, to ensure brain structures with a particular risk to change in the deep space environment do not put astronauts and the mission in jeopardy [76].

## 5. Neurological Monitoring Technologies in the Deep Space Environment

To compare the neurological monitoring technologies described in this review, Table 1 summarizes their characteristics, applications on the ground, and use in spaceflight. All scanner-based techniques such as MRI, CT, and PET are large devices that logistically cannot be launched onboard space vehicles or stations at this time. Although there have been proposals for the development of MRI devices for use in space, one has yet to be developed [79,80]. EEG appears to be an excellent option for clinical applications and neurological research in deep space. In terms of functional neuroimaging specifically, fNIRS is also an excellent option for neuroimaging during space missions.

While any scanner-based imaging (MRI, PET, SPECT, CT) appears to be difficult to integrate onboard a space station, fNIRS may be an excellent alternative to a technology such as fMRI. This device trades a degree of image resolution for portability and long-term monitoring capabilities [43]. A fNIRS device onboard a deep space station would enable studying astronaut performance through direct measures of an astronaut’s brain activity in real time [81]. Although fNIRS has not yet been used in spaceflight, there is the potential for its use on a station such as the Lunar Gateway. A rationale for its use was provided in a NASA report, mostly supported by the argument that it was portable enough for spaceflight [82]. In recent years, more portable and smaller versions of fNIRS have been commercialized [82]. Another key concern is its ability to retain reliability over long-term exposure to microgravity and radiation environments. Although NASA studies suggest that better designed headgear will reduce the variability in collected data, more work must be carried out to test fNIRS in deep space radiation environments [82].

In the clinical setting, measurement of cerebral oxygenation and autoregulation can be performed with fNIRS, although other applications of fNIRS are also transferable to deep space [44,83]. Technology has been developed that combines the use of an fNIRS device with that of an EEG for simultaneous brain monitoring. Although this integrated EEG/fNIRS monitoring system is also low-cost and lightweight, it is fairly new, and it has not been tested for the purposes of spaceflight [45]. Conventional fNIRS is more established in analog and pre- and post-flight studies, though its ability to analyze different brain signals simultaneously may prove to be a useful tool for deep space research in the future [44,82].

Ultrasound was another potential consideration for deep space neurological monitoring, however it should not be prioritized over EEG and fNIRS. EEG can support studies on the effects of head injury in the way ultrasound is currently being used on the ISS, and fNIRS is an excellent option for measuring brain functionality. Ultrasound cannot generate images of the brain, although it can be used to measure changes in fluid pressure in the brain, as well as cerebral blood flow and velocity [52]. Although this has its uses in neurological studies centered around the impacts of microgravity, these kinds of data are only indirectly related to brain function [48].

EEG is a brain monitoring option that is portable, has a wide range of clinical and research applications, and has even been used in LEO onboard the ISS [84]. EEG’s medical applications are more aligned with concerns about the impact of the deep space environment. Monitoring technologies have been made available to support EEG in real-time longitudinal monitoring of both brain health and vital signs (i.e., NeuroCatch™ platform). The existence of monitoring platforms for EEG greatly widens the ability for its use as a medical tool as well as a research technology [37,38]. Among its diagnostic capabilities, EEG can support the detection of brain tumors, and in-flight brain injury during long flights with exposure to high LET radiation [10,24,25]. Space research enabled by EEG could improve brain monitoring as well as support the development of protective measures against the deep space environment. Moreover, this imaging device could support space-related research on the effects of sleep deprivation, mental health ailments, cognitive performance, SANS, extended isolation, heightened radiation exposure, and prolonged microgravity exposure.

In addition to monitoring capabilities, technologies such as NeuroCatch™ are also able to assess brain activity related to motor function, language, and functional regions of the brain, such as the temporal lobe [85,86,87]. By being able to evaluate motor function robustly and accurately in networks of the brain related to upper extremities or establish differences in brain activity in different brain areas, EEG has the capacity for both medical and research applications onboard platforms such as the Lunar Gateway [85,87].

## 6. Conclusions

While there are many studies conducted on Earth using spaceflight analogs or investigations collecting data in LEO aboard the space shuttle or the ISS, the impact of the deep space environment on brain function has not been characterized so far. The Lunar Gateway program will provide the first opportunity to conduct studies that will help reduce the neurological risks of space missions beyond LEO. Several technologies were identified as good candidates for in situ data collection, whereas others are more suited for pre/post-flight studies. Space neuroscience is currently an embryonic research field that will lead to advances in our understanding of human brain function, and hopefully lead to space countermeasures as well as terrestrial applications. Furthermore, many technologies have benefited from improvements in portability or performance due to constraints of spaceflight, and it is expected that it will be similar for brain function monitoring techniques. Overall, expanding the reach of human spaceflight will not only enrich our knowledge of the universe, but will also lead to concrete benefits to healthcare on Earth.

## 7. Methodology

A search of PubMed, Scopus, and Google Scholar was conducted as well as forwards and backwards searches of the citations of included articles to help procure relevant information on neuroimaging and brain monitoring techniques. The following string search was used: (Neuroimaging OR Monitoring) AND (Deep Space OR Spaceflight).

## Figures and Tables

**Table 1:** Neurological monitoring device characteristics and applications.

Imaging Devices	Analog or Pre/Post-Flight Studies	Utilized in LEO	Good Candidate for Deep Space	Metrics Measured by Device	Major Terrestrial Clinical Uses	Major Terrestrial Research	Typical Size (Volume and Weight)
**EEG**	YES	YES	YES	Brain activity as waveform patterns of spectral content	Brain tumors, head injury, brain dysfunction (encephalopathy, encephalitis), stroke, sleep disorders	Brain tumor, brain damage, head injury brain dysfunction, stroke, and sleep disorder research	**Device Dimensions** = 8.91 × 6.11 × 2.38 cm **Weight** = 0.078–0.096 kg
**Ultrasound**	YES	YES	YES	Structural measurements, middle cerebral artery velocity (MCAv)	Cardiovascular measurements, cerebrovascular examination, and pediatric neuroimaging	Cerebrovascular research and pediatric neuroimaging research	Typical Device: **Dimensions** = 34.0 × 35.5 × 15.0 cm **Weight** = 3.1 kg Portable Device: **Dimensions** = 17.2 × 5.4 × 4.2 cm **Weight** = 0.120 kg
**MRI: Structural**	YES	NO	NO	Volumes, surface areas, regional thickness, and other measurements that characterize the structural features of the brain	Demyelinating diseases, dementia, cerebrovascular disease, infectious diseases, Alzheimer’s disease, epilepsy, edema, tumor, infarction, inflammation, infection, hyperacute or chronic hemorrhage	Alzheimer’s, epilepsy, cranial nerves, Parkinson’s disease, schizophrenia, trauma, tumors, multiple sclerosis, and surgical applications	**Length** = 186 cm, **Weight** = 7000 kg
**MRI: Functional**	YES	NO	NO	BOLD signal, functional connectivity	Maps brain activity from sensory, motor, cognitive, and emotional tasks.	Functional connectivity, multiple sclerosis, Alzheimer’s disease, epilepsy, brain tumors, stroke, traumatic brain injury, behavioral, and psychological performance.	**Length** = 186 cm, **Weight** = 7000 kg
**MRI:** **Diffusion**	YES	NO	NO	Fractional anisotropy (FA), mean diffusivity (MD), axial diffusivity (AD), and radial diffusivity (RD)	Cerebral infarction (DWI), white matter deformation by tumors, and dementia	Surgical planning, microstructure imaging, white matter connectivity, neurodevelopmental changes	**Length** = 186 cm, **Weight** = 7000 kg
**fNIRS**	NO	NO	YES	Oxygen concentrations, Hb concentrations, blood-oxygen level-dependent signal (BOLD signal), and functional connectivity	Global cerebral function, cerebral oxygenation, and autoregulation in patients with stroke and traumatic brain injury in critical care.	Functional connectivity, fetal alcohol syndrome, mental disorders, behavioral and psychological performance.	**Dimensions** = 16.2 × 12.5 × 6.0 cm **Weight** = 0.900 kg
**Computed Tomography**	NO	NO	NO	Volumes, surface areas, regional thickness, and other measurements that characterize the structural features of the brain	Detecting infarction (stroke), tumors, calcifications, hemorrhage, and bone trauma, and angiography of cerebral arteries.	Brain injury identification and treatment, tumor identification and treatment, structural abnormalities, stroke	**Dimensions** = 199 × 89 × 222 cm **Weigh** = 2100 kg
**Positron Emission Tomography**	NO	NO	NO	Metabolism rate (glucose metabolism)	Alzheimer’s, dementia, epilepsy, movement disorders, stroke, and cardiovascular disorder tumors	Oncology, Alzheimer’s, seizure, schizophrenia, substance abuse, mood disorders, and other psychiatric conditions, psychological disorders, and processes	**Dimensions** = 200 × 228 × 168 cm **Weight** = 2100 kg
